# RNA Polymerase II Elongation at the Crossroads of Transcription and Alternative Splicing

**DOI:** 10.4061/2011/309865

**Published:** 2011-09-20

**Authors:** Manuel de la Mata, Manuel J. Muñoz, Mariano Alló, Juan Pablo Fededa, Ignacio E. Schor, Alberto R. Kornblihtt

**Affiliations:** ^1^Laboratorio de Fisiología y Biología Molecular, Departamento de Fisiología, Biología Molecular, y Celular, IFIBYNE-CONICET, Facultad de Ciencias Exactas y Naturales, Universidad de Buenos Aires, C1428EHA Buenos Aires, Argentina; ^2^Friedrich Miescher Institute for Biomedical Research, 4002 Basel, Switzerland; ^3^Department of Biology, Institute of Biochemistry, ETH Zurich, 8093 Zurich, Switzerland

## Abstract

The elongation phase of transcription lies at the core of several simultaneous and coupled events leading to alternative splicing regulation. Although underestimated in the past, it is at this phase of the transcription cycle where complexes affecting the transcription machinery itself, chromatin structure, posttranscriptional gene regulation and pre-mRNA processing converge to regulate each other or simply to consolidate higher-order complexes and functions. This paper focuses on the multiple processes that take place during transcription elongation which ultimately regulate the outcome of alternative splicing decisions.

## 1. Introduction

Regulation of gene expression was originally conceived as a hierarchy of steps linked together on a time scale and physically separated in different cell compartments in accordance with the central dogma of biology. This concept has long been abandoned, with a significant accumulation of evidence describing an extensive network of events, encompassing transcription, mRNA processing, chromatin regulation, and posttranscriptional gene regulation, which take place simultaneously and in a mutually regulated or coupled manner [1, 2]. Distinctions between complexes and processes governing gene expression have been blurred to a large extent, adding complexity to the ever-increasing fraction of genes subjected to alternative promoter usage, alternative splicing (AS) (>90% genes), alternative polyadenylation, editing, and posttranscriptional gene silencing by small RNAs [3, 4]. Additionally, this complexity takes a new dimension when studied in the context of chromatin and its regulation upon gene expression (for reviews, see [5–7]). This paper will focus on the main features of coupling between transcriptions elongation and splicing, and its implications on AS regulation.

## 2. The Benefits of Coupling

Initial visualization of Drosophila-embryo nascent transcripts by electron microscopy, showed that splicing can occur cotranscriptionally [8]. This was later directly demonstrated for the human dystrophin gene [9], which spans 2400 kb and can take 16 hr to complete transcription. More recently, a quantitative study of the c-Src and fibronectin mRNAs compared chromatin-bound and nucleoplasmic RNA fractions. There, it was shown that most introns are excised efficiently in the chromatin-bound fractions, with a gradient of cotranscriptional splicing efficiency decreasing from promoter-proximal to promoter-distal introns, that is, the direction of transcription [10]. 

One implication of the cotranscriptional nature of splicing is that the two processes can be coupled. In a broad sense, coupling implies that the involved processes can happen efficiently only as the result of their combined action, even for processes that are constitutive and non-regulated. For instance, whereas both transcription and splicing can take place independently at low efficiency as *in vitro* reactions, it is only *in vivo* or in coupled *in vitro* systems where maximal efficiency can be achieved [11–16]. Cotranscriptional processing is necessary to allow for coupling between transcription and splicing, although it does not necessarily guarantee it. There are examples of both cotranscriptional splicing that seems to be uncoupled, as well as purely posttranscriptional splicing [17–20]. Noteworthy, the consequences of the different types of splicing can be considerable; whereas cotranscriptional splicing can be regulated by mechanisms dependent on transcription, postranscriptional splicing can be subjected to additional regulatory mechanisms linked to events downstream of transcription (e.g., RNA export) [20]. However, cotranscriptional splicing seems to predominate for most introns in mammalian genes [10, 17, 20–23] pointing at an evolutionary conserved role in allowing for coupling of transcription and splicing. Cotranscriptional splicing is more efficient than posttranscriptional splicing by driving nascent pre-mRNAs to the association with spliceosome components [24, 25] and splicing regulatory factors, such as serine/arginine-rich (SR) proteins [22]. This allows for different levels of regulation of AS and prevents backhybridization of the nascent pre-mRNA to the DNA template strand, which can cause genome instability [26, 27]. Even in the case of posttranscriptional splicing, coupling with transcription can be determinant for AS regulation. Since pre-mRNA splicing is a multistep reaction, it is possible that commitment to splicing takes place cotranscriptionally during early splice-site recognition, while completion of the splicing reaction occurs posttranscriptionally [20, 28, 29], consistent with the fact that introns are not necessarily removed in the exact order that they are transcribed [17, 30–32]. This mechanism, which can be viewed as a cotranscriptional commitment rather than a cotranscriptional catalysis, tends to apply largely to splicing and not to other RNA-processing events like capping and cleavage/polyadenylation [33–37]. Intermediate scenarios are also possible, with both splicing commitment and catalysis taking place cotranscriptionally but not following a strict 5′ to 3′ direction of intron removal [10, 38].

Another implication of cotranscriptional splicing is that it allows for a bidirectional coupling of the two processes [1, 20, 39, 40]. For instance, the splicing machinery can reciprocally affect transcription in different ways by either stimulating transcriptional elongation [41, 42], transcriptional initiation [43, 44] and, as recently shown in yeast, by imposing a transient pausing checkpoint around the 3′ end of introns and on terminal exons [45, 46]. This bidirectional feedback might in turn reinforce splicing efficiency, conferring important advantages for gene expression. Nevertheless, reciprocal coupling might not be a widespread general phenomenon considering that elongation kinetics seems to be independent of splicing in some model genes [47]. This highlights the possibility that specific exon-intron architectures and/or cis-acting sequences might be required for reciprocal coupling to occur.

Despite all the seemingly clear advantages of cotranscriptional over posttranscriptional splicing, particularly in allowing for coupling, the true proportions of these two modes of splicing in mammals still await to be determined on a genome-wide scale. It will be interesting to use global approaches to answer this question, which might have profound implications in reorienting our current research and on our understanding of the regulation of AS.

## 3. Early Discoveries

**Figure 1:**
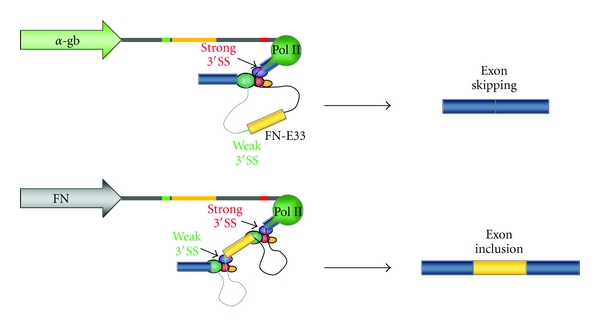
Promoters affect alternative splicing. *α*-globin/FN hybrid minigenes under the control of different promoters, used in transient transfections of mammalian cells in culture to assess inclusion levels of the alternatively spliced E33 (EDI or EDA) cassette exon (dark yellow). Inclusion level with the FN promoter is >10-fold higher when compared to the *α*-globin promoter.

In our current view, the regulation of AS is the result of the combined action of splicing factors acting on splicing enhancers and silencers, regulatory secondary structure motifs of mRNAs, and the coupling with RNA polymerase II (Pol II) transcription [22, 48–51]. An early indication for coupling was the finding that promoter identity affects splicing decisions independently of the strength of the promoter, opposing the classical view whereby promoters are limited to affect transcription levels ([52–56], for review, see [57]). Different promoters, such as those of the *α*-globin and fibronectin (FN) genes, were shown to induce a 10-fold difference in inclusion of the human FN alternative exon 33 (E33, also referred to as EDI or EDA) when driving its expression from reporter minigenes in transiently transfected mammalian cells [52, 53] (Figure 1).

One implication of the promoter effect on AS is that splicing factors could regulate AS through promoters. Cell-specific AS events could then arise from cell-specific promoter occupation rather than from the differential abundance of ubiquitous splicing factors. Under physiological conditions, promoter architecture could then control AS through the differential occupation by different transcription factors. Supporting this hypothesis, evidence shows that transcriptional activators and coactivators, with different effects on Pol II, indeed affect AS differentially [58, 59].

## 4. The Kinetics of Coupling

Transcription appears to influence pre-mRNA splicing through at least two independent modes: by kinetically coupling the processing reactions, where the rate of Pol II elongation influences the outcome of the alternative events [60, 61], and by physically and functionally recruiting mRNA processing factors to the transcription machinery, in particular to Pol II's carboxyterminal domain (CTD) [13, 62–65]. In fact, recruitment of splicing factors to sites of transcription is dependent on RNA Pol II CTD [66] and deletion of the CTD impairs capping, cleavage/polyadenylation, and splicing of the b-globin transcript [34]. Here we provide a view of the kinetic mode of coupling, although accumulated evidence supports that both modes can operate simultaneously in a nonmutually exclusive manner (for reviews on the recruitment mode of coupling, see [2, 65, 67]). 

**Figure 2:**
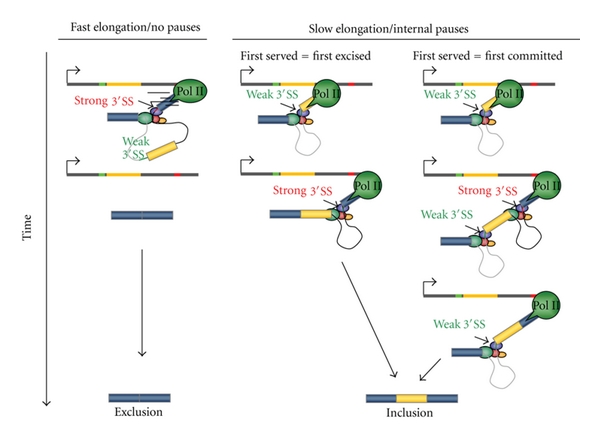
Alternative models for the “first come, first served” mechanism of splice site selection. (a) Fast elongation promotes usage of the stronger downstream 3′ splice site. (b) Slow elongation causes preferential excision of the upstream intron (first served = first excised). (c) Slow elongation causes commitment to E33 inclusion via recruitment of splicing factors (first served = first committed). Both introns are excised individually and in an order that is independent of elongation. (Based on [38].)

It should be pointed out that transcription itself is a complex multistep and regulated process, organized as a transcription cycle, with each step subjected to extensive regulation [68]. However, it is mainly at the elongation step of transcription where most of the connections between the transcription and splicing machineries actually occur. A role for Pol II elongation on AS had been suggested before the finding of the promoter effect [69] and was later supported by several lines of evidence. Eperon et al. [69] showed that, in contrast to *in vitro* conditions, ongoing RNA synthesis *in vivo* affects the potential secondary structure of long—but not that of short—RNA substrates, which in turn affects splicing, pointing at a kinetic link between transcription and splicing. Additional evidence came from experiments in which RNA Pol II's local pausing caused by elements inserted into the tropomyosin gene, promoted higher inclusion of tropomyosin exon 3 [60]. However, more conclusive evidence for a role of elongation on AS regulation was shown in a series of reports demonstrating that several factors globally impacting Pol II elongation also affect AS. (i) Replication of AS reporter minigenes greatly stimulates FN E33 inclusion, and is counteracted by trichostatin A (TSA), a potent inhibitor of histone deacetylation considered to drive chromatin into an “open” state. This suggested that replication conveys a more compact chromatin structure to the template, thus slowing elongation and leading to higher E33 inclusion [70]. (ii) Drugs known to inhibit elongation, such as DRB [58, 70], flavopiridol, or camptothecin [38], favor E33 inclusion. (iii) Transcriptional activation by VP16, a factor that promotes both initiation and elongation, decreases E33 inclusion while Sp1, acting only on initiation, has no effect on E33 inclusion [58]. (iv) The presence of the SV40 transcriptional enhancer near a promoter stimulates Pol II elongation and provokes a 3–10-fold reduction in FN E33 inclusion independently of the promoter used [71]. (v) Slow mutants of RNA Pol II increase FN E33 inclusion in human cells, affect AS of the endogenous gene ultrabithorax (Ubx) in Drosophila, and modulate the inclusion of an artificially created alternative exon in yeast [61, 72]. (vi) DNA-damage signaling triggered by UV irradiation affects the AS of fibronectin, caspase 9, Bcl-x, and other human genes by inducing hyperphosphorylation of Pol II CTD and blocking Pol II elongation [73]. These data are in agreement with a “first come, first served” model for regulation of AS [74] (Figure 2). One interpretation of this model is that slow elongation favors the removal of the intron upstream of an alternative cassette exon before removal of the downstream intron. Alternatively, slow elongation would favor recruitment of splicing factors to the upstream intron before the downstream intron is synthesized. Once commitment to include the exon is achieved, the order of intron removal becomes irrelevant (Figure 2). The latter interpretation is supported by recent evidence showing a preferential removal of the intron downstream of the FN cassette exon 33 before the upstream intron has been removed [38]. Most importantly, whereas cis-acting mutations and trans-acting factors that upregulate E33 inclusion alter the relative order of intron removal, elongation slowdown also induces higher E33 inclusion without affecting the order of intron removal, suggesting that slow elongation favors commitment to exon inclusion during spliceosome assembly [38]. In light of these findings, “first served” would not be equivalent to “first excised” but to “first committed,” in agreement with the observed preferential cotranscriptionality of spliceosome recruitment rather than catalysis.

The control of elongation on AS is not restricted to a few cases. Recent data confirms and extends the findings of elongation control on AS using a global approach based on AS microarray profiling [48]. This study demonstrates that a variety of conditions that impact Pol II elongation, including drug treatments (DRB and camptothecin), Pol II mutants that inhibit elongation (slow and CTD phosphorylation Pol II mutants), and cellular stress (UV irradiation, know to impact Pol II elongation), globally affect both the mRNA levels and AS of a significant number of the genes. Moreover, the largest statistically significant fraction of affected genes showed a concomitant decrease in steady-state mRNA levels with increase in exon inclusion levels, coincident with many of the examples previously reported [60, 61, 70, 72, 73, 75, 76]. Nevertheless, there was also a smaller but significant number of genes displaying decreased mRNA levels with decreased exon inclusion levels [48], consistent with the idea that inhibition of Pol II elongation can lead to increased exon inclusion as well as increased exon skipping depending on the underlying splicing regulatory mechanism involved in each case [55, 61]. Pol II's elongation-dependent changes in AS regulation also display a high preference to modulate the expression levels of genes involved in RNA metabolism, including pre-mRNA splicing factors and other RNA binding proteins. Interestingly, about one-third of those genes contain AS events that introduce a premature termination codon (PTC) when spliced into the mature mRNA, subsequently leading to nonsense-mediated mRNA decay (NMD) [48]. This represents another example of evolutionarily conserved elongation-coupled events, acting together to coordinate the levels or RNA binding proteins with their steady-state mRNA levels.

## 5. Elongation Links to Chromatin

The promoter effect, together with the kinetic, physical, and functional coupling modes of transcription and AS, immediately shifted the attention to other factors thought to be restricted to transcriptional regulation, such as the chromatin structure. It soon became clear that the unanticipated complexity of splicing regulation could not be explained solely based on the current models, which lacked a clear connection to *in vivo* situations. Chromatin is the natural substrate upon which transcriptional regulation acts *in vivo*, and major discoveries have recently pointed at chromatin structure and post-translational histone modifications as key regulators of AS.

The chromatin role on AS regulation is broad, involving both direct (elongation-independent) interactions with the splicing machinery and effects on AS through changes in transcription elongation. Here, we concentrate on the later, although recent findings on the direct roles of chromatin on splicing have significantly changed our view of how exon-intron architecture is achieved by intimately linking nucleosome to exon structure ([77–84], for reviews, see [5–7]. In fact, genome-wide mapping of nucleosome positioning on exons at the DNA level may shed light on one of the most striking puzzles in the field of splicing, that is, how does the splicing machinery recognizes, with high fidelity, short exons (on average 150 bp) “floating” in a “sea” of introns (on average 5.4 kbp), an exon-intron architecture typical of vertebrate genes [85, 86]. Notably, the average size of a mammalian exon is similar to the length of DNA wrapped around a nucleosome, suggesting a conserved function for the nucleosome in exon definition [79, 80, 82]. According to the exon definition model, originally postulated by S. M. Berget [87], the spliceosome and auxiliary factors achieve this recognition by preferentially assembling on 3′ and 5′ sites paired across exons and not across introns (i.e., not following an intron definition mode of recognition, typical in lower eukaryotes like yeast). This favors exon recognition and acts as a selective force for short exon size. As described below, nucleosome positioning on exons may help in exon definition by creating roadblocks for Pol II elongation that provide longer time for cotranscriptional recognition of splice sites in the nascent pre-mRNA.

## 6. Chromatin Structure in Coupling

**Figure 3:**
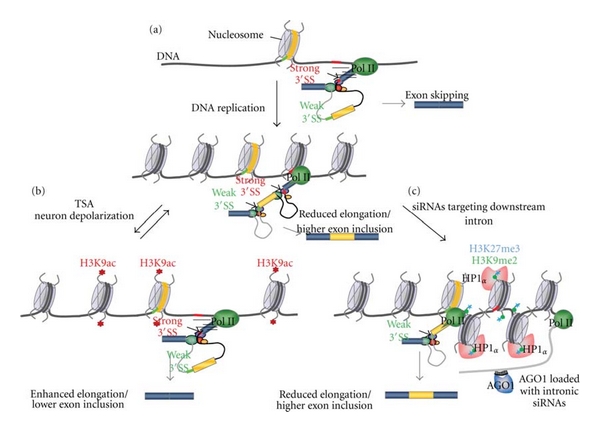
Chromatin couples elongation to alternative splicing. (a) Early evidence for a role of chromatin on splicing: replication affects alternative splicing. Loose nucleosome assembly (as in transiently transfected reporter minigenes) gives rise to low inclusion of the alternative exon (yellow) into the mature mRNA. After replication, nucleosome organization becomes more compact, promoting much higher inclusion of the alternative exon. (b) Depolarization of neuronal cells or treatment with TSA triggers intragenic histone acetylation and looser nucleosome compaction which in turn causes skipping of the alternative exon (yellow). (c) Model for TGS-AS. Transfection with siRNAs targeting the intron downstream from the alternative exon (yellow) promotes dimethylation and trimethylation of H3K9 and H3K27 (green and blue marks, resp.), triggered by siRNA's guide strand entering a silencing complex containing AGO1. HP1*α* is recruited and the resulting condensed chromatin structure generates roadblocks to Pol II elongation, causing higher inclusion of the alternative exon according to the kinetic coupling model.

A role for chromatin in the coupling of elongation with AS was first suggested with the finding that two copies of the same adenovirus genome, either unreplicated or replicated in the same nucleus, gave rise to different alternatively spliced RNAs [57]. It was then speculated that the chromatin organization acquired after replication was more compact, with a subsequent reduction in Pol II elongation rates and more time to assemble splicing complexes at a suboptimal upstream 5′ splice site, thus favoring its use compared to the downstream 5′ splice site. This was confirmed by the finding that FN E33 inclusion is sensitive to the replication-mediated chromatinization status of the reporter plasmid [58, 70] (Figure 3(a). See above). Both in plants and humans genes, RNA Pol II distribution correlates with nucleosome deposition with preferential accumulation at exons relative to introns [79, 80, 83, 88], consistent with nucleosomes acting as barriers that locally modulate RNA Pol II density by inducing its pausing [89, 90]. This could in turn modulate splicing efficiency, in agreement with RNA Pol II being more highly enriched at alternatively spliced exons than at constitutive ones [88]. Nucleosome density also varies according to splice site strength, with stronger positioning of nucleosomes in included alternatively spliced exons than excluded ones [80] and stronger positioning at exons defined by weaker splice sites [81, 82]. Consistent with these results, overexpression of the ATPase-dependent chromatin-remodeling complex SWI/SNF subunit Brm in human cells, induces accumulation of Pol II with a modified CTD phosphorylation pattern on regions encoding variant exons of the CD44 gene and causes increased inclusion of these exons into mature mRNA [91]. 

## 7. Histone Modifications in Coupling

Histone modifications have emerged as major regulators of AS by either impacting the coupling of elongation with AS or by direct means such as recruiting splicing factors to the nascent pre-mRNA [7]. An indication for a role of histone modifications in the coupling of elongation with AS was the observation that treatment of cells with the histone deacetylase inhibitor TSA induces skipping of the alternatively spliced fibronectin E33 and the neural cell adhesion molecule (NCAM) exon 18 [58, 75, 76]. In a more physiological context, depolarization of neuronal cells increases H3K9 acetylation and H3K36 methylation locally around the alternatively spliced exon 18 of NCAM which correlates with increased exon skipping [76] (Figure 3(b)). Interestingly, no changes in histone acetylation were observed at the NCAM promoter, suggesting that this reversible effect may be due to an intragenic and local modulation of the RNA Pol II elongation rate [76]. For instance, acetylated and open chromatin would induce fast Pol II elongation and skipping of the alternative exon. In line with these observations, targeting of an intronic sequence downstream of the alternatively spliced E33 of fibronectin with small interfering RNAs induces local heterochromatinization and increased E33 inclusion without changes in general transcription levels [75] (Figure 3(c)). In addition, inhibition of histone deacetylation, DNA methylation, H3K9 methylation, and downregulation of heterochromatin protein 1*α* (HP1*α*) abolish the siRNA-mediated effect on exon E33 splicing [75], suggesting a role of these modifications in AS regulation. Considering the multiple evidence for the involvement of chromatin in siRNA effects on splicing, this mechanism has been referred to as TGS-AS for transcriptional gene-silencing-regulated AS [92] (Figure 3(c)).

Several other epigenetic modifications, including DNA methylation, may also directly or indirectly act via histone modifications to affect splice site decisions [83, 93, 94]. This adds to the additional mechanism involving direct physical crosstalk between chromatin and the splicing machinery via an adaptor complex [7, 95, 96].

## 8. Conclusions and Outlook

The fact that transcriptional elongation is largely connected to pre-mRNA processing and particularly to AS regulation has boosted extensive efforts to understand its mechanisms and physiological implications. The link to chromatin seems to be the natural way in which elongation and splicing are truly engaged and might indeed explain the poorly understood mechanisms by which tissue- and cell-type-specific AS patterns are established, propagated, and maintained. Although some cell- and tissue-specific differences in expression of constitutive splicing factors have been reported [97], it is tempting to speculate that, in analogy to the histone code mechanisms used to specify gene expression, a histone-based system may also encode information that specifies cell- and tissue-specific AS patterns. As recently proposed, this would provide an epigenetic memory for splicing decisions likely to be heritable during proliferation and susceptible to modification along differentiation, without the need for major changes in the AS rules at each step of differentiation [7]. Nevertheless, it still remains to be determined whether the effects of histone modification on RNA processing are heritable and therefore authentic epigenetic modifications or whether they are just transient modulators.

Given the bidirectional nature of coupling, it is conceivable that splicing might reciprocally feed back on the chromatin structure via affecting the transcription machinery. In fact, besides recruiting splicing regulators such as SR proteins or U2 snRNP subunits [25, 64], Pol II can interact with histone modifiers such as the histone 3 lysine 36 (H3K36) methyltransferase Set2 [98], known, for example, to regulate histone deacetylation and prevent inappropriate initiation within the body of transcribed genes [99]. This interesting possibility would build up on the notion that transcription, chromatin, and splicing are intimately dependent on each other.

Future directions of the field will most likely aim at a more comprehensive view on the histone modifications role in AS. Histone modifications must be comprehensively mapped on a genome-wide scale in multiple cell types and tissues and compared to global AS patterns. New candidate players will be most likely be studied, such as noncoding RNAs recently shown to be involved in heterochromatin structure and associated with AS regulation [100, 101]. Ultimately, a better understanding of the multiple links between transcription and splicing will head the way to decipher the complexity of gene expression both in physiological and pathological conditions.
